# Missed Opportunities for HIV Prevention in Perinatal Care Settings in the United States

**DOI:** 10.3389/frph.2021.680046

**Published:** 2021-06-14

**Authors:** Lealah Pollock, Marliese Warren, Judy Levison

**Affiliations:** ^1^National Clinician Consultation Center, Department of Family and Community Medicine, University of California, San Francisco, San Francisco, CA, United States; ^2^Department of Obstetrics and Gynecology, Baylor College of Medicine, Houston, TX, United States

**Keywords:** hiv prevention, pregnancy, breastfeeding, HIV self-testing, partner testing, pre-exposure prophylaxis

## Abstract

Universal opt-out HIV screening in pregnancy is an essential intervention toward eliminating perinatal HIV transmission in the US. However, it fails to identify pregnant people who are HIV negative at the time of testing but are at ongoing risk for HIV acquisition. Those of us involved in caring for women living with HIV are acutely aware of the many diagnoses of HIV that might have been prevented if only a partner had been tested for HIV or preexposure prophylaxis (PrEP) had been offered to a patient. This perspective article will review current recommendations and evidence-based interventions to evaluate missed opportunities for HIV prevention in US perinatal care settings. We identified three barriers to implementation of HIV prevention strategies during pregnancy and breastfeeding: (1) HIV risk for women is underestimated and poorly defined in clinical practice; (2) Partner testing is challenging and implementation studies in the US are lacking; and (3) PrEP remains underutilized. In March 2020, the National Perinatal HIV Hotline convened a group of clinicians and researchers specializing in perinatal HIV care to a case-based discussion of missed opportunities in perinatal HIV prevention. From our review of the literature via PubMed search as well as expert opinions gathered in this discussion, we make recommendations for addressing these barriers.

## Introduction

Universal HIV testing in pregnancy is an essential step in preventing perinatal HIV transmission. However, testing only the pregnant patient fails to identify people at risk for HIV acquisition during pregnancy and breastfeeding and misses opportunities to interrupt sexual and perinatal transmission of HIV (1). Risk for HIV acquisition per receptive vaginal condomless sex act increases substantially during pregnancy and in the postpartum period (2, 3). In addition, seroconversion during pregnancy and breastfeeding carries a high risk of HIV transmission to the baby and is an ongoing obstacle to the goal of eliminating perinatal HIV transmission in the US. A PubMed search of articles from 2006–2021 was conducted using key words pre-exposure prophylaxis and HIV and (women or pregnancy or pregnant or conception or preconception or postpartum or breastfeeding) as well as pre-exposure prophylaxis and (peri conception or peri-conception or periconception). We reviewed the citations in relevant articles in order to identify additional literature for inclusion. This perspective article will review current recommendations and evidence-based interventions to evaluate missed opportunities for HIV prevention in US perinatal care settings. We will also present opinions generated from a gathering of perinatal HIV experts convened in March 2020.

## Evidence-Based Interventions

Recommended and tested interventions generally fall into two categories: increasing provision of preexposure prophylaxis (PrEP) during pregnancy and breastfeeding, and offering HIV testing to sexual partners of pregnant people.

### Provision of PrEP During Pregnancy and Breastfeeding

PrEP is a highly effective HIV prevention method in which an HIV-negative individual takes antiretroviral medications in order to prevent HIV acquisition. The only medication that is currently approved for HIV prevention among cisgender women in the United States is tenofovir disoproxil fumarate 300 mg-emtricitabine 200 mg (TDF-FTC), in the form of a daily oral pill. Other medications and routes of administration are under investigation.

TDF and FTC have been shown to be safe during many years of use as part of an antiretroviral regimen for pregnant women living with HIV and, more recently, as PrEP for HIV-negative women (4, 5). When used during breastfeeding, breast milk concentrations of tenofovir are low, and infant plasma concentrations are <1% of pediatric therapeutic levels (6, 7). Despite being highly efficacious and safe, PrEP remains underutilized during pregnancy and breastfeeding (8).

A “PrEP care continuum” has been proposed as a framework to understand PrEP implementation and dissemination in at-risk populations (9). The first step in the PrEP care continuum is generally defined as PrEP awareness, which has three components: identifying individuals at highest risk for contracting HIV, increasing HIV risk awareness among those individuals, and enhancing PrEP awareness (10). This framework is particularly helpful in thinking about PrEP implementation in populations with low HIV risk awareness, such as pregnant and breastfeeding individuals.

#### Providers Unaware of HIV Risk

In 2017, the Centers for Disease Control (CDC) (11) identified indications for PrEP use by heterosexually active men and women, including HIV-negative women not in a monogamous relationship with a recently tested HIV-negative partner who also have at least one of the following risk factors: infrequent condom use with one or more partners of unknown HIV status who are known to be at substantial risk of HIV infection, in an ongoing sexual relationship with an HIV-positive partner, or infection with syphilis or gonorrhea diagnosed or reported in the last 6 months. Using these criteria, Fruhauf and Colemen (12) estimated that 10% of their pregnant population in Baltimore were eligible for PrEP. However, this list is somewhat unwieldy for the busy practicing clinician.

#### Women and Partners Unaware of HIV Risk

Studies in the US suggest that women may underestimate their HIV risk and the HIV risk status of their male partners (13, 14). Women may be unaware that their male partners have risks for HIV. Partner characteristics that present a risk for HIV include concurrent partnerships with women and/or men, untreated sexually transmitted infections (STIs), injection drug use, prior incarceration, and undisclosed or undiagnosed HIV infection (15). Relying on a biological marker of HIV risk, such as diagnosis of a bacterial STI, also fails to identify a significant number of women who will later acquire HIV (16). In one survey of African American women, age over 35, being recently homeless, being on Medicaid, and last sex partner characteristics (crack cocaine use and being a transactional sex partner) were more strongly associated with a new HIV diagnosis than any individual risk factor (17).

A history of trauma, including intimate partner violence (IPV) and substance use, including non-injection substance use, are additional risk factors for HIV (18, 19). One study showed women experiencing IPV were more worried about getting HIV in the next 6 months, but their PrEP awareness and intentions were the same as women without these experiences (20). Engaging in transactional sex in exchange for drugs as well as loss of inhibitions can be seen with both injection and non-injection drug use. Substance use clinics have therefore been suggested as ideal sites for offering PrEP (21).

#### Patient Awareness of PrEP and Provider Willingness to Prescribe

Studies of at-risk women have demonstrated a low public awareness of PrEP, although this awareness appears to be increasing over time and likely varies by location, with 6–44% of women reporting having heard of PrEP (13, 22, 23). Even using existing guidelines to identify women at risk of acquiring HIV, there are huge gaps in implementation. An analysis of nationwide insurance claims data from 2017 found that only 6–12% women diagnosed with gonorrhea or syphilis were tested for HIV and none of these patients were prescribed PrEP (24). Studies assessing PrEP awareness specifically among pregnant patients and prenatal providers are lacking at this time. Interviews with clinicians documented in two qualitative studies have elucidated conflicting perceptions about who should be responsible for prescribing PrEP (25, 26). Many primary care physicians believe that PrEP prescription is in the purview of specialists, while many specialists see it as the responsibility of primary care clinicians. In a survey of family planning providers in 2015, only about one-third answered basic knowledge questions about PrEP correctly (27). Some clinicians said they would consider prescribing if patients specifically requested PrEP, which assumes knowledge and high motivation on the part of patients (25, 26). The larger view of assessing all women for periconception, pregnancy, and postpartum risk has yet to be embraced on a national scale in the US and other countries (28).

### HIV Testing for Partners of Pregnant and Breastfeeding People

US guidelines recommend that partners of pregnant women undergo HIV testing when their status is unknown. The goal is to facilitate linkage to care for partners with HIV and guide a discussion about prevention (8, 11). The challenges of following this recommendation have been highlighted in implementation studies in US settings.

There are two primary approaches to testing partners of pregnant women –offering testing for male partners, not tied to the prenatal HIV testing of the pregnant partner; or offering counseling, testing, and disclosure with a trained counselor to both partners as a couple. Both of these approaches can be carried out either in the clinic or at home. In Sub-Saharan Africa, study participants have expressed a variety of preferences about where and how HIV testing should occur; pregnant patients and their partners often have different preferences (29, 30).

#### Partner HIV Testing

We could identify no studies looking at home-based testing among pregnant women and their partners in the US. In Kenya and Uganda, home-based self-testing and home-based testing administered by trained personnel both resulted in two- to three-times higher uptake of male partner testing and couples' testing and higher rates of HIV status disclosure than inviting male partners to the clinic for HIV testing. However, linkage to care after HIV testing at home remains a challenge (30–33).

In one clinic in Chicago, two-thirds of participants were interested in knowing their partner's status and three-quarters of them believed their partner would like to know his status (34). However, only 39% of participants reported that their partner had insurance coverage for medical care or a primary care provider.

Another study invited HIV-negative pregnant women to bring their male partner to their next prenatal visit for a free HIV test, but only 20.6% of invited males underwent HIV testing (35). The authors found that decisions about testing were driven by perceptions about fidelity, male partner autonomy, fetal safety, ease of testing, and recency of prior HIV testing.

#### Couples' HIV Testing

Couples' HIV testing has been evaluated for its ability to increase condom use within a partnership, and is effective, but studies in the US have primarily been done outside of the context of pregnancy and prenatal care (36). In one urban academic antenatal care setting in the US, couples who received couples HIV testing and counseling reported a very high level of acceptability and increased ease in having conversations around safe sex (37). However, only 8% of eligible couples consented for the study. The most common reasons for declining participation were difficulty bringing a partner in for testing, including scheduling conflicts for the partner and the partner not being available or interested, and low perceived risk for HIV infection.

### Addressing Community and Structural-Level Risk for HIV

In 2018, Blacks/African Americans made up 13% of the female population but accounted for 58% of new HIV diagnoses among women (38). Individual risk behaviors cannot explain the dramatic racial disparities in HIV rates (14, 15, 39). Non-Hispanic Black women are more likely to have concurrent sexual partners and to perceive their partners to be nonmonogamous. However, they are also more likely to use condoms than White women, suggesting that other social and structural factors likely contribute to HIV acquisition risk (40).

Multiple authors have highlighted the role that racial segregation, higher community baseline HIV and STD prevalence, poverty, gender inequality, mass incarceration, lack of access to healthcare, and racism play in driving racial disparities in HIV prevalence (14, 15, 39–41). Ojikutu (39) concludes that women at high risk may be “hidden in plain sight,” to be found if clinicians would pay greater attention to sociodemographic factors than individual sexual behaviors. Assessing socioeconomic/contextual factors that increase HIV risk may be more helpful than individual behavioral risk factors or sex partner characteristics (14, 15, 17). However, these social and structural factors that continue to drive the HIV epidemic among women must primarily be addressed with structural interventions (42). While offering PrEP to pregnant patients who engage in transactional sex or have substance use disorder is important, offering economic opportunities, stable housing, non-stigmatizing mental health care, comprehensive syringe services programs, and access to substance use treatment may be far more effective in reducing their risk for HIV and improving health overall. Moreover, as women's HIV vulnerability is directly linked to community-level HIV prevalence and HIV viral load, interventions to decrease HIV stigma in the population and decrease bias and discrimination in health care will help to mitigate this vulnerability (43).

### Best Practices and Future Directions

The National Perinatal HIV Hotline (www.nccc.ucsf.edu) hosted roundtable discussion in 2020, *Preventing Maternal HIV Transmission during Pregnancy and Breastfeeding*, that coincided with the Conference on Retroviruses and Opportunistic Infections (CROI). Attendees were clinicians, HIV researchers, federal funders, and community members who discussed current practices and future directions. The discussion is summarized below.

#### HIV Risk Assessment: Pregnant Person's Risk

Participants identified prenatal care visits as an opportunity to discuss each patient's social and reproductive history, including previous STI diagnoses. This discussion can be framed as a routine part of care to ensure the pregnant person's and baby's health. Discussing HIV as one of many relevant infections and conditions can help normalize the condition, particularly when providers avoid using stigmatizing language. Providers can also routinely ask pregnant people whether they have new sexual partners without making assumptions about relationships or partner concurrency.

#### HIV Risk Assessment: Partner Risk

Among HIV providers, asking about partner HIV status and encouraging partner testing is often routine. However, in a general prenatal/clinical setting, it is not standard practice. The current American College of Obstetrics and Gynecology (ACOG) prenatal form includes questions that ask about patient and partner history of hepatitis, tuberculosis, and herpes as well as patient history of STIs including gonorrhea, chlamydia, human papilloma virus, and syphilis. The group suggested that ACOG include a question about the HIV status of sexual partner(s). If partner status is unknown, providers could offer partner HIV testing and discuss PrEP.

#### Couples' HIV Testing

Participants identified barriers to couples' HIV testing in prenatal care in the US, including wariness about deferring HIV testing of the pregnant person in order to test both partners simultaneously. One proposed solution focused on partner testing by linking pregnant people with partners of unknown HIV status to a PrEP coordinator and comprehensive services for partners (e.g., HIV and STI testing, vaccines like Tdap and influenza, and linkage to primary care).

#### Secondary Distribution of HIV Self-Tests for Partners

As discussed above and also noted by roundtable participants, HIV self-test dispensation within prenatal care is ongoing broadly in East and Southern Africa and seems to be acceptable to patients and their partners. In the UK, self-testing kits are available and free (44). HIV self-testing should be explored as a strategy for partner testing in the US.

#### Universal Education About HIV Prevention and PrEP

Participants noted that assessing risk in a low prevalence population remains an issue for evaluating PrEP eligibility. One proposed solution was universal education about HIV risk and PrEP. Anyone who requests PrEP should receive it, regardless of the clinician's assessment of risk. Participants in the group noted that pregnant people may not wish to discuss their HIV risk behaviors but may respond to being offered PrEP. Additionally, personal risk factors and behaviors often change over time. There are times when a woman might not be sexually active and might not want to remain on PrEP continuously but would like the option to return to PrEP.

## Discussion

We identified three areas that contribute to missed opportunities for HIV prevention in pregnancy and breastfeeding: (1) HIV risk awareness among women is low and HIV risk for women is challenging to identify and define in clinical practice; (2) Partner testing is far from routine and implementation studies in the US are lacking; and (3) PrEP remains underutilized among women, especially during pregnancy and breastfeeding. Utilizing our review of the literature, the views and opinions shared during the 2020 roundtable discussion, and our own experience and perspectives, we will share next steps and opportunities for addressing each of these gaps.

### HIV Risk for Women Is Challenging to Identify and Define in Clinical Practice

Individual factors that should alert clinicians to HIV risk include a recent (and not so recent) STI diagnosis; infrequent condom use with one or more partners of unknown HIV status, especially within a high-prevalence sexual network; a history of intimate partner violence; engaging in transactional sex; substance use disorder and/or substance use associated with sex; having a partner with HIV without consistent virologic suppression; and having a partner with any of the factors listed here. Questions about these risks could be routinely assessed in perinatal care settings, using prenatal intake questionnaires or checklists. However, these checklists have been challenging to implement, partly because standardized HIV risk assessment tools for cis-gender women in the US haven't been developed. Also, many of these factors, especially those involving partner characteristics, are often unknown to pregnant people themselves.

One question that is easy to implement is: “Are any of your sexual partners living with HIV?” This question has emerged as an important screening question for all people seeking preconception, pregnancy, and postpartum care, both in the literature and in our roundtable discussion (1, 27, 34). Even if the response is “I don't know my partner's HIV status,” the question may lead to a discussion about partner testing and PrEP.

Being at risk is a function of both environment (e.g., living in a community with high underlying HIV incidence) and individual exposure to risk (e.g., having condomless sex with a partner with untreated HIV) (45). While individual- and partner-level risk factors for HIV are important to understand and assess, community- and structural-level factors play a very large role in individual HIV risk. Clinicians should understand the contextual risks of HIV acquisition, especially among low income or homeless women, women living in the South, and women of color, but should avoid profiling individual women based on poverty, geography, or race. Being aware of the HIV prevalence where one is practicing is crucial and could potentially be a point of discussion when talking to patients about their individual HIV risk (41). Interventions that target inviduals should be grounded in principles of equity and evaluated based on their impact on health disparities, but structural-level interventions are needed in order to combat structural-level health determinants. Interventions aimed at reducing inequities and racism in policing, criminal justice, education, economic opportunity, physical and mental health care, and housing will likely have very real impacts on reducing HIV infections and should be included and evaluated as part of efforts to eliminate HIV transmission (42, 43, 46).

Population-based risk factors could be utilized to develop standardized risk assessment tools, none of which have been developed or validated for cis-female populations in the US. However, standardized risk assessment tools have their own drawbacks: they can be challenging to validate in a low-prevalence region or population, they are not generalizable to other populations beyond the one in which they were validated, and they are likely to miss individuals who are high-risk but “screen out” by the tool (45). Additionally, development of risk assessments often occurs without community input and risks exacerbating rather than decreasing bias and stigma by creating a “profile” of a patient at risk (39). Clinicians and policy makers need to talk to community members, both those living with HIV and those who are at-risk, and incorporate their input when developing risk assessments. Discussing “risk” may not be the right approach at all. Dazón Dixon Diallo has pointed out that HIV “vulnerability” might better capture the life conditions and structural factors that create an opportunity for HIV acquisition (47).

### Partner Testing Is Challenging and Implementation Studies in the US Are Lacking

Very few studies in the US have assessed attitudes toward or effectiveness of interventions to offer HIV testing to partners of pregnant people. The studies that have been done have demonstrated a desire for partner testing but have also highlighted low uptake of testing and multiple barriers to testing (34, 35, 37).

The fragmentation of the US healthcare system is an unfortunate barrier to partner HIV testing. How do you create an entry in the EMR for a male partner seen in a prenatal care setting? Who is responsible for tracking and following up on results? Outside of couples' testing, how and when and by whom does disclosure occur? Who pays for partner HIV testing? System changes, such as single payer healthcare, would allow partner and couples' HIV testing to support the health of pregnant people, their infants, and their partners. Despite these challenges, the perinatal period presents a potential opportunity to engage partners in their own healthcare by framing it as being in service to the birthing person and infant.

While HIV incidence, HIV stigma and attitudes toward HIV testing are likely different in the US than in other countries, and also differ among subpopulations within the US, we can still gain important knowledge and insights from studies of male partners testing in sub-Saharan Africa. In particular, the concept of offering different options for male partner testing and the increased uptake of home-based testing are important considerations to apply to future studies in the US. The US is not dispensing HIV self-tests to pregnant people for secondary distribution due to concerns of suicide/self-harm or lack of linkage to HIV care among those who test HIV-positive (48–50). However, the potential to use self-testing as a strategy to reach partners of pregnant and breastfeeding people was highlighted by our roundtable participants.

The CDC encourages the implementation of HIV self-testing programs to meet the ambitious goals of the federal Ending the HIV Epidemic (EHE) initiative^1^. Based on the success of the eSTAMP study (51), two EHE jurisdictions in California began utilizing self-testing kits and found that fears of self-harm and patients lost to follow up were not realized and were outweighed by the benefits of privacy. Test counselors and patient navigators were able to remain connected to their clients and all patients who received a preliminary positive HIV test received confirmatory tests and were successfully linked to care as needed (52).

### PrEP Remains Underutilized Among Women, Especially During Pregnancy and Breastfeeding

National organizations, such as the CDC and ACOG, should more strongly endorse the use of PrEP in pregnancy and breastfeeding, beyond its use in serodifferent couples. These organizations can also help develop and promote tools to assist prenatal care providers in assessing HIV risk, promoting partner HIV testing, and offering PrEP to all pregnant and breastfeeding women who are interested. The ACOG obstetric patient record forms should include questions about partner HIV status and partner HIV risk and could include prompts for offering PrEP. Electronic medical record technology could be used to streamline the process of ordering baseline labs, ordering PrEP, planning timing of follow up labs and appointments, and obtaining approval for financial coverage of PrEP. Excitingly, current EHE efforts have eliminated the financial barrier to PrEP for people without insurance coverage^2^.

Part of increasing PrEP uptake is also increasing community-level PrEP awareness, including awareness that PrEP can be used as an HIV prevention tool during pregnancy and breastfeeding. Community-based education programs can reach women who may not come to clinic and plant the seed for people before they become pregnant (5). Additionally, educational materials in clinic waiting rooms or examination rooms, and public messaging on television, radio, and social media can be used to disseminate information about PrEP more widely.

The Department of Health and Human Services (DHHS) Panel on Treatment of Pregnant Women with HIV Infection and Prevention of Perinatal Transmission and the World Health Organization (WHO) agree that all viable HIV prevention options, including PrEP, should be encouraged for women at risk for HIV, especially during pregnancy and breastfeeding, given the increased risk of HIV acquisition during pregnancy and the potential for perinatal transmission with maternal seroconversion during pregnancy (8, 53). The DHHS Panel cites many indications for PrEP, including simply feeling at risk for HIV. While not the only method of HIV prevention, PrEP offers women a tool they can control to protect themselves without having to negotiate with a partner (54). Combined with routine opt-out HIV testing and assessment of partner HIV status, offering PrEP during pregnancy and breastfeeding has the powerful potential to eliminate perinatal HIV transmission. US clinicians interested in learning more about prescribing PrEP to their patients can call the PrEPline toll free and speak with an expert clinician consultant: nccc.ucsf.edu.

## Data Availability Statement

The datasets presented in this study can be found in online repositories. The names of the repository/repositories and accession numbers can be found below: https://nccc.ucsf.edu/wp-content/uploads/2017/08/2020NatlPeriHotline_CROIRoundtableSummary_10.08.pdf.

## Author Contributions

All authors listed have made a substantial, direct and intellectual contribution to the work, and approved it for publication.

## Conflict of Interest

The authors declare that the research was conducted in the absence of any commercial or financial relationships that could be construed as a potential conflict of interest.

## Footnotes

^1^ https://www.cdc.gov/hiv/testing/self-testing.html

^2^ https://www.hiv.gov/federal-response/ending-the-hiv-epidemic/prep-program
